# A Rapid Gas-Chromatography/Mass-Spectrometry Technique for Determining Odour Activity Values of Volatile Compounds in Plant Proteins: Soy, and Allergen-Free Pea and Brown Rice Protein

**DOI:** 10.3390/molecules26134104

**Published:** 2021-07-05

**Authors:** Anika Singh, Yuan Shi, Perrine Magreault, David D. Kitts, Maciej Jarzębski, Przemysław Siejak, Anubhav Pratap-Singh

**Affiliations:** 1 Natural Health and Food Products Research Group, Centre for Applied Research & Innovation (CARI), British Columbia Institute of Technology, 4355 Mathissi Pl, Burnaby, BC V5G 4S8, Canada; anika_singh@bcit.ca; 2 Food, Nutrition and Health, Faculty of Land & Food Systems, 2205 East Mall, The University of British Columbia, Vancouver, BC V6T 1Z4, Canada; yuannnshii@gmail.com; 3 Cursus Ingénieur Agroalimentaire, 65, Rue de Saint-Brieuc, CS 84215 Rennes, France; perrine.magreault@hotmail.fr; 4 Department of Physics and Biophysics, Faculty of Food Science and Nutrition, Poznań University of Life Sciences, Wojska Polskiego 38/42, 60-637 Poznań, Poland; maciej.jarzebski@up.poznan.pl (M.J.); przemyslaw.siejak@up.poznan.pl (P.S.)

**Keywords:** plant protein, volatile aroma compounds, odour threshold value, pea protein, rice protein, soy protein

## Abstract

Plant-based protein sources have a characteristic aroma that limits their usage in various meat-alternative formulations. Despite being the most popular plant-based protein, the allergenicity of soy protein severely restricts the potential adoption of soy protein as an animal substitute. Thereby, allergen-free plant-protein sources need to be characterized. Herein, we demonstrate a rapid solid-phase-microextraction gas-chromatography/mass-spectrometry (SPME-GC/MS) technique for comparing the volatile aroma profile concentration of two different allergen-free plant-protein sources (brown rice and pea) and comparing them with soy protein. The extraction procedure consisted of making a 1:7 *w*/*v* aqueous plant protein slurry, and then absorbing the volatile compounds on an SPME fibre under agitation for 10 min at 40 °C, which was subsequently injected onto a GC column coupled to an MS system. Observed volatile concentrations were used in conjunction with odour threshold values to generate a Total Volatile Aroma Score for each protein sample. A total of 76 volatile compounds were identified. Aldehydes and furans were determined to be the most dominant volatiles present in the plant proteins. Both brown rice protein and pea protein contained 64% aldehydes and 18% furans, with minor contents of alcohols, ketones and other compounds. On the other hand, soy protein consisted of fewer aldehydes (46%), but a more significant proportion of furans (42%). However, in terms of total concentration, brown rice protein contained the highest intensity and number of volatile compounds. Based on the calculated odour activity values of the detected compounds, our study concludes that pea proteins could be used as a suitable alternative to soy proteins in applications for allergen-free vegan protein products without interfering with the taste or flavour of the product.

## 1. Introduction

The solid-phase microextraction (SPME) is a simple, sensitive, robust, reliable technique based on analyte diffusion that combines the advantages of both static and dynamic headspace for qualitative analysis of volatiles [1]. On the other-hand, gas-chromatography/mass-spectrometry (GC/MS) techniques are commonly used techniques for detailed organic compounds detection and analysis in foods and food components at the molecular level. The combination of SPME and GC/MS (SPME-GC/MS) is a well-established and commonly employed method for several detection procedures, including identification of odours in different samples from wastewater treatment to food quality assessment [2,3] or even degradation of plastic materials [4] and natural environment control [5]. A notable development of these methods has focused on combining GC/MS with an olfactometric detection (GC/MS-O) to separate and detect odourant components [6,7,8]. Furthermore, many comparative studies focusing on the relationship between GC/MS odour and aroma identification and subjective individual assessment of the product attractiveness confirmed that volatile compounds and aroma strongly influence consumer preferences and decisions [3,7,9]. Therefore, GC/MS and GC/MS-O studies are essential investigation tools for evaluation of any edible product’s desirability in terms of the off-odours.

Formulating novel food products using alternative plant protein sources requires effective methods to remove or mask volatiles derived from natural, green, beany attributes, often described by consumers as off-flavours. Numerous studies have attempted to reduce the most potent odour-active volatiles in protein sources, n-hexanal, a lipoxygenase-derived degradation product of linoleic acid, very commonly identified in soybean [10]. To characterize the volatile aroma profile of different plant proteins, improvements in the extraction of volatiles are needed.

A plant-based diet is associated with good human health and environmental sustainability. Soy protein is the most commonly consumed plant-based protein source consumed by vegans. Soy protein is associated with significant decreases in serum cholesterol and low-density lipoprotein (LDL) cholesterol concentrations [11]. The Food and Drug Administration (FDA) authorized the use of health claims for soy protein to reduce the risk of coronary heart disease on labelling of food products containing soy protein [12]. However, soy protein is also a recognized food allergen [13], while on the other hand, rice and pea proteins are generally regarded as hypoallergenic, and several studies have highlighted the nutritional and health benefits associated with the consumption of these proteins. Increasing dietary intake of rice protein has been associated with reduced serum cholesterol levels and overall lowering of lifestyle-related diseases [14]. The protein content of the pea ranges from 21% to 23% [15], and has been identified to provide multiple health benefits that include lowering plasma cholesterol and plasma triglyceride levels, thus protecting against atherosclerosis [16].

In this study, we used hexanal, 2-nonanone and hexanol as standards for aldehydes, ketones, and alcohols, respectively, which can be quantified. Hexanal, which has a green and grassy aroma [17], is a product of enzymatic or autoxidative decomposition of unsaturated fatty acids and is the most abundant aldehyde detected in peas [18]. Similarly, 2-nonanone with a fresh, green, weedy and earthy smell was present in all protein sources. Hexanol was another dominant volatile compound identified in most plant-based proteins and is known to have a herbaceous, woody and green aroma [19].

**Figure 1:**
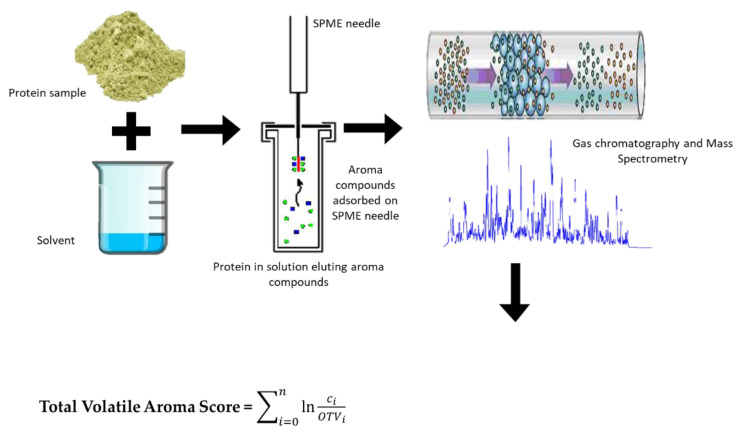
Application of a rapid SPME-GC/MS technique to recover and identify a complex mixture of aroma volatile compounds from proteins.

This work demonstrates the application of a rapid SPME-GC/MS technique to recover and identify a complex mixture of volatile aroma compounds that can be used as signatures for the volatile aroma composition of a pea, rice, and soy proteins (the schematic idea is presented in Figure 1). This information will help better understand the characteristic off-flavour chemistry in these plant-based protein sources and possible strategies for removing them using novel food processing technologies. Furthermore, comparing pea and rice proteins with a volatile aroma profile generated from soy protein could also aid in choosing the right protein source for allergen-free alternatives to conventional vegan formulations employing soy protein.

## 2. Results

### 2.1. Volatile Aroma Extraction and Quantification Optimization

The fully quantified concentrations of hexanal, 2-nonanone and hexanol recovered from the three plant-protein isolates used herein are provided in Table 1. Other compounds were only semi-quantified, wherein the aldehydes, ketones and alcohols were expressed as equivalent concentrations of hexanal, 2-nonanone and hexanol, respectively, while other compounds were represented as equivalents of hexanal. All semi-quantified volatile compounds with characteristic odours and retention times, and corresponding odour activity values and odour threshold values are given in Table S1, for soy, allergen-free pea and brown rice proteins.

### 2.2. Aldehydes

**Figure 2:**
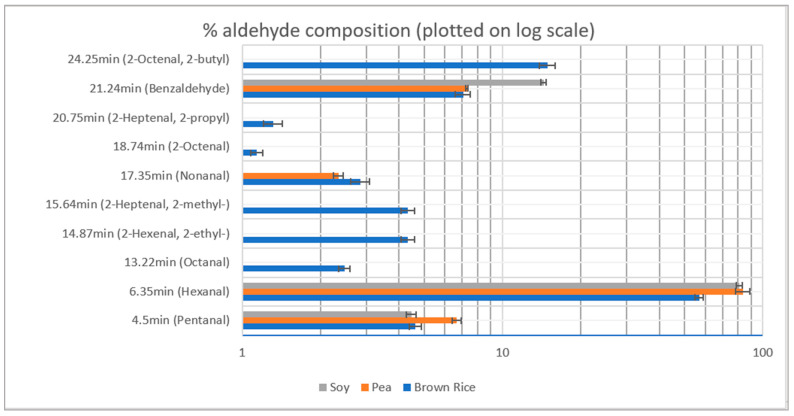
% aldehyde composition in the plant protein isolates.

The total aldehyde concentration of allergen-free brown rice and pea protein were observed to be 40,250 ± 3938 and 1359 ± 321 ppb equivalents of hexanal, respectively, while that of soy protein was observed to be 1998 ± 201 ppb equivalents of hexanal. Thus, Pea protein was found to be a suitable alternate to soy protein if the aldehyde composition is required to be decreased. Figure 2 shows the % contribution of individual aldehydes identified along with their retention times. Hexanal, pentanal and benzaldehyde were prominent aldehydes in all the three plant protein isolates. Brown rice contained a lower concentration of each of the above three aldehydes; however, it also contained other aldehydes including 2-butylocten-2-al, at a relative concentration greater than pentanal and benzaldehyde, and others including octanal, 2-ethylhexen-2-al, 2-methyl-hepten-2-al, 2-propylhepten-2-al and 2-octenal.

### 2.3. Ketones

**Figure 3:**
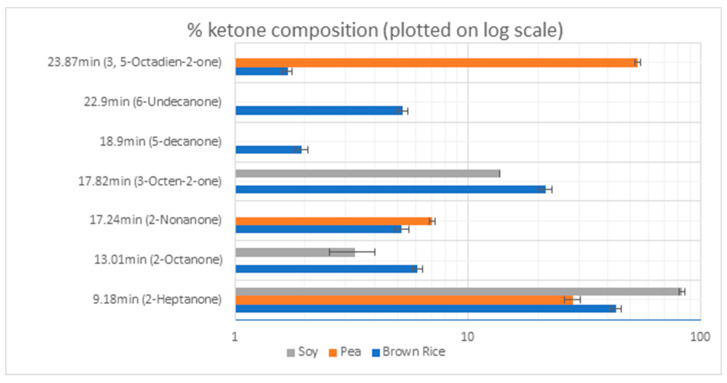
% ketone composition in the plant protein isolates.

The total ketone concentration of allergen-free brown rice and pea protein was observed to be 1801 ± 263 and 91 ± 18 ppb equivalents of 2-nonanone, respectively, while soy protein was observed to be 87 ± 11 ppb equivalents of 2-nonanone. Figure 3 shows the % contribution of most important ketone compounds identified in the three plant protein powders. Pea protein was found to be close to soy protein in volatile composition. 2-heptanone was a primary ketone identified in all three proteins, whereas brown rice and soy contained 2-octanone and 3-octen-2-one. Pea protein contained a high concentration of 3,5-octadien-2-one and its structural isomers, whereas brown rice also contained it in an appreciable amount. 2-nonanone was detected in appreciable amounts in both allergen-free pea and brown rice proteins but was absent in soy protein.

### 2.4. Alcohols and Other Compounds

The total alcohol concentration of brown rice protein was 4852 ± 458 equivalents of hexanol. Total alcohol concentration of soy protein was only 40 ± 9 equivalents of hexanol in form of 1-octen-3-ol, while pea proteins contained no alcohol compounds. 1-octen-3-ol and 1-pentanol were major compounds (>20%) identified in brown rice proteins, with appreciable quantities (>5%) of hexanol, heptanol, octanol and 3-nonen-2-ol.

Amongst other compounds, furans were a major source of volatile compounds. 2-pentyl furan was found in large quantities in soy and pea proteins (2492 ± 199 and 638 ± 49 ppb equivalents of hexanal, respectively, for soy and pea proteins), whereas 386 ± 30 ppb equivalents of hexanal of 2-n-butyl-furan were observed in brown rice protein.

Brown rice protein was also found to contain appreciable amounts of alkanes (octane: 652 ± 41 ppb equivalent of hexanal), alkenes (5-Undecene: 1410 ± 297 ppb equivalent of hexanal), sulfides (dimethyl trisulfide: 303 ± 40 and 273 ± 43 ppb equivalent of hexanal, respectively), and carboxylic acid (pentanoic acid: 225 ± 47 ppb equivalent of hexanal).

### 2.5. Comparison of Odour Activity of Soy and Other Allergen-Free Protein Sources

Based on the proposed volatile aroma score, brown rice had the highest total volatile aroma score of 102.79. On the other hand, pea and soy proteins had similar total volatile aroma scores of 24.43 and 28.89, respectively; nevertheless, pea protein was the least active odour and a suitable alternative to allergenic soy protein.

## 3. Discussion

**Figure 4:**
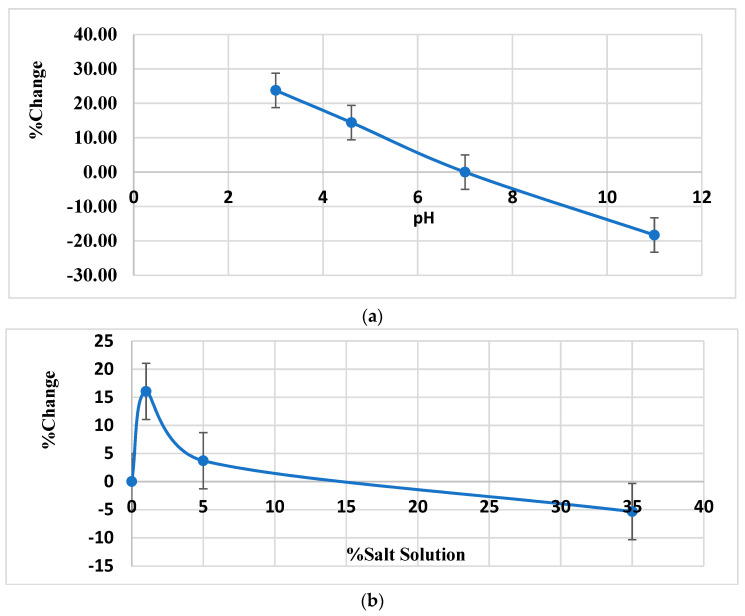
(**a**) percent change to the normalized area under the curve as compared to pH 7, (**b**) percent change to the normalized area under the curve as compared to distilled deionized water (0% salt solution).

We observed that diluting different protein powders in distilled water was sufficient to recover volatiles when performed at lower pH, thus pointing to the importance of water in perceiving food flavour [20]. However, increasing the pH from 3 to 11 gradually decreased volatiles’ recovery due to lower resolution attributed to poor SPME performance (Figure 4a). Furthermore, the addition of 1% NaCl, also increased recovery of volatiles; whereas adding a greater amount of salt (e.g., 5–35% *w*/*v*) was found to be less effective (Figure 4b) due to lower protein solubility and hence lower recovery of associated organic compounds [21].

Even though GC/MS has a high sensitivity for volatile detection, the matrix effect presented by individual proteins influences the accuracy of volatile compound quantification [22]. However, we observed that the calibration of soy and pea proteins could be changed interchangeably, which is probably attributed to both having a high concentration of proteins as the major compounds. As a result, the physical properties were the same, which produced similar partitions as the binding between volatiles and proteins are predominantly derived through hydrophobic interactions [23].

Aldehydes and ketones dominated the composition of volatiles recovered from the three different protein sources used in this study, which is in agreement with results obtained by other researchers [24,25]. Furthermore, aldehydes were found to be the most abundant volatile compounds recovered that used different protein sources. These volatiles are generated in plant products by either enzymatic or autoxidative decomposition of fatty acids, mainly from precursors linoleic and linolenic acids [26].

The presence of hexanal in both soy and pea proteins is a product of linoleic acid oxidation and has been described by other researchers to contribute to the green and beany off-flavour reported in peas and soy proteins [27]. Other researchers have reported a grassy, green and “fat-like” odour associated with pea and soy proteins, respectively [19]. Moreover, hexanal, which has a very low odour threshold (about 5 ppb) in water [28] is regarded as a potent recognition volatile due to it being detected at very low concentrations [29]. Pentanal also has a low odour threshold of 12ppb and is known for its green and milky-like odour [25]. Benzaldehyde, another trace aldehyde (<1% of total aldehyde concentration) detected, has a higher odour threshold of about 13 ppb [30]. Lipid oxidation, derived from linoleic acid substrates [31], and Strecker degradation from an amino acid such as phenylglycine [32] in vegetable protein hydrolysates [33], are common pathways for the production of benzaldehyde in soy and pea protein.

Other aldehydes previously reported in plant proteins, but not detected in this study, included 2-nonenal, decanal and undecanal. It is feasible that the extraction efficiency of these compounds in aqueous solvents was limited due to especially strong interactions with the protein matrix [34].

It is of interest that the detection of 2-heptanone and 2-nonanone, previously attributed to off-flavours described in pungent cheeses [35], were recovered from pea protein; thus potentially contributing to the off-flavour of pea protein.

1-Octen-3-ol, another product of lipid oxidation, derived from lipoxygenase activity of linoleic acid precursors, was recovered in soy and brown rice protein. The only alcohol compound existing in soy protein sample was 1-Octen-3-ol, and it contributed to 37.03% of the alcohol compound in brown rice. Other researchers have characterized 1-octen-3-ol to have a low odour threshold (e.g., 0.001 ppm) with a distinct mushroom odour [24].

In addition, pea protein samples did not contain any alcohol. This contrasted the alcohol profile derived from soy protein, which was higher in 1-hexanol, along with a small proportion of 1-heptanol.

Furans, formerly attributed to the grassy–beany and green flavour in pea protein are an additional class of volatile aromas recovered from the three different plant proteins studied [36]. Some alkanes and alkenes were also identified in all protein sources studied; however, these volatiles do not have a major role in generating off-flavours. Indeed, pure alkanes are considered tasteless and nearly odourless.

The odour activity values of various compounds identified in the three plant-protein sources are first reported herein and will be used for future studies that focus on flavour reengineering of plant-based food products. While other researchers have used volatile aroma scores, the natural logarithmic of these scores were reported in this study, and a sum of the natural logarithmic scores was used to quantify the total volatile aroma profile. This value will be used in future studies as a numerical indicator of the volatile aroma intensity, which shall allow use in numerical optimization studies that are focused on reducing the off-flavour intensity of plant-protein isolates.

Based on our research, what is more interesting is that even if the SPME partition coefficients vary, our approach of taking logarithmic values of the ratio of concentration and odour threshold helped us reduce the effect of variation in coefficient.

(1) $$Actual\;Concentration\;\left(c_{i}\right)=Semi\_quantified\_concentration\times SPME\_partition\_factor{\;}_{\;}$$

(2) $$\ln\frac{c_{i}}{OTV_{i}}=\ln\left(\frac{Semi\_quantified\_concentration}{OTV_{i}}\right)+\ln\left(\frac{SPME\_partition\_factor}{OTV_{i}}\right)$$

Log of the *SPME partition factor* will be constant across multiple samples with different volatile profiles, and thus, the factor log (*semi-quantified concentration*/*OTV*) can still be used for comparison purposes.

The simplified approach divides possible aroma compounds into different categories and uses just a single standard for the analysis of a particular set of compounds. This study used three standards for three categories of compounds—ketones, alcohols and aldehydes. The accuracy of the total volatile aroma score increased as the number of categories increased. Proper care must be taken in choosing the particular categories of different volatile compounds.

It must also be mentioned that despite the representation of different compounds with respect to a single or a few sets of standards for each category of compounds, the total volatile aroma score is still reliable as this calculation involves a logarithmic transformation of the ratio between the semi-quantified concentration and the odour threshold value of the compounds. The score is only affected for those compounds whose concentrations are orders of magnitude away from the odour threshold value; these value parameters can be obtained using a discriminative sensory study, or chemically using an e-nose apparatus.

The methodology proposed in this research can be applied across the field of optimization of complex systems, wherein the volatile aroma profile score can be used as a numerical factor demonstrating the behavior of systems during food product design and development.

Nevertheless, our investigations indicate that pea protein was better than brown rice as an allergen-free alternative to replace soy proteins and can be used for developing food products and applications that require minimal impact on the aroma profile of the product.

## 4. Materials and Methods

### 4.1. Materials

Pea protein (Daiya Foods, Burnaby, BC, Canada), isolated soy protein and organic brown rice powder (Nuts.com, Cranford, NJ, USA) were used as examples of plant protein sources. All chemicals were purchased from VWR (Mississauga, ON, Canada). Distilled deionized (DD) water was purified through a Milli-Q purification system (Millipore Corp., Bedford, MA, USA). All inorganic chemicals were of analytical reagent grade and were purchased from Sigma Aldrich (Oakville, ON, Canada). Internal standard hexanal-d12, and other standards (hexanal, hexanol and 2-nonanone) were purchased from Sigma Aldrich (Oakville, ON, Canada).

### 4.2. Methods

A solid-phase microextraction, followed by gas chromatography-mass spectrometry (SPME-GC/MS) was used to extract volatile aromatic compounds present in plant protein samples for quantification. To extract the volatiles, 1 g of plant protein powder was dissolved in 7 mL distilled deionized water. Volatile compounds were recovered on a 1 cm − 50/30 μm divinylbenzene/carboxen/poly-dimethylsiloxane (DVB/CAR/PDMS) Flex SPME fibre over a period of 10 min, as increasing the time to 30 min resulted in less than 10% change in total area under the curve (Figure S1). The flex fibre had been conditioned at 250 °C before the extraction of samples in a sample vial heated at 40 °C with 10 min agitation. The extraction of headspace volatiles was performed in triplicate from the same sample lot for each protein sample.

The SPME fibre was transferred to a GC injection port and analysis was performed using a GC auto-sampler (6890 N Agilent), coupled to an MS detector (5963 N Agilent). Analytes were desorbed from the SPME fiber into a DB-WAX 122-7062 capillary column (60 m × 0.25 mm ID, 0.25 μm film thickness) to perform the chromatographic separation. SPME fiber was injected into a pulsed-spitless injector, set at 250 °C, for a 5 min period, for the desorption to complete. Helium was used as the carrier gas at a flow rate of 1.4 mL/min, and additional pressure of 30 psi was applied for 5 min. The temperature program was initially set at 40 °C for 4 min, followed by an increase of 3 °C/min to 150 °C, and then 25 °C/min to reach 230 °C, after which it was kept at 230 °C for 7 min. An Agilent 5973 quadrupole mass spectrometer was operated in the electron ionization mode at 70 eV, with quadrupole temperature was set at 150 °C, and a scan ranged from 40 to 500 *m*/*z*. Data were collected using Agilent ChemStation software (standard MSD version) and volatiles recovered were searched using both NIST (v. 02) and Wiley (v. 138) libraries (Palisade Corp., Newfield, NY, USA).

Standard curves of hexanal, 2-nonanone, and hexanol were generated and used for the aldehyde, ketone and alcohol quantification separately. Quantification of aromatic volatile components was determined using a response factor and area under the curve for each compound in the chromatogram. Known standards were chosen based on preliminary SPME-GC/MS analysis results of pea samples. Hexanal, 2-nonanone and hexanol were selected to represent the aldehyde, ketone and alcohol groups, respectively. The standard compounds stock was prepared as follows: 10,000 mg/L hexanal, 100 mg/L 2-nonanone, and 100 mg/L hexanol. The pea protein sample was spiked with 1, 2, 3, 4, 5 μL of stock standard and 5 μL of internal standard hexanal-d12 was used to generate the standard curve for quantification (Figure S2).

The volatile aroma score of all the compounds was determined based on the volatile aroma concentration and the proposed Equation (3):

(3) $$\mathrm{Total}\;\mathrm{Volatile}\;\mathrm{Aroma}\;\mathrm{Score}=\sum_{i=0}^{n}\ln\frac{c_{i}}{OTV_{i}}$$

where, *c_i_* is the concentration of the *i*th compound (ppb), and *OTV_i_* is the odour threshold value of the *i*th compound (ppb).

### 4.3. Statistical Analysis

Each experiment was performed on individual proteins in triplicate, with each result analyzed in triplicate, and the results were expressed as the mean ± standard deviation (*n* = 9).

## 5. Conclusions

Odour is one critical factor which has a strong impact on customers’ food choices and eating habits. Application of the SPME-GC/MS technique for rapid detection and comparing the volatile aroma profile could speed up new food product development in food technology and manufacturing. Knowing the odour profile of the food ingredients helps to adjust and optimize conditions during food processing. It must be pointed out that soy protein, allergen-free pea protein, and allergen-free brown rice protein are in great interests as emulsions and food product stabilizers. For that reason, it is important to study the odour profile of proteins, to avoid potential interactions in the complex systems. A methodology was proposed to quantify the odour profile based on SPME-GC/MS data by considering the odour threshold value. Brown rice was found to be the most odour-active, while soy and pea proteins had similar total aroma score. Such an approach is expected to find utilization in multi-factor optimization problems involving different characteristics of a biological sample. Furthermore, the study concludes that pea proteins might be a suitable allergen-free alternative to soy proteins, to minimize impacts on the off-flavour of the product.

## Figures and Tables

**Table 1:** Fully quantified concentration of the standard compound (hexanal, 2-nonanone, and hexanol) in plant protein extracts.

Concentrations	Pea	Brown Rice	Soy
Hexanal (in ppb) ^1^	1138.00 ± 297.30	22,590.24 ± 1643.70	1621.71 ± 159.69
2-Nonanone (in ppb) ^1^	6.382 ± 0.62	94.02 ± 12.38	n.d.
Hexanol (in ppb) ^1^	n.d.	102.04 ± 9.30	n.d.

^1^ Values expressed as the mean ± standard deviation.

## Data Availability

All data related to this study are presented in this publication.

## Supplementary Materials

The following are available online. Figure S1: Comparison of chromatogram resolution of pea protein isolates with a 10 min equilibration time (black) and a 30 min equilibration time (blue), Figure S2: Calibration curves generated for hexanal, 1-hexanol and 2-nonanone in pea and soy protein isolates (calibration curves in red are for pea, and that in blue are for soy), Table S1: Volatile profile of soy protein, allergen-free pea protein, and allergen-free brown rice protein.
