# Supplementary material for: A Rapid Gas-Chromatography/Mass-Spectrometry Technique for Determining Odour Activity Values of Volatile Compounds in Plant Proteins: Soy, and Allergen-Free Pea and Brown Rice Protein
Source: Molecules. 2021 Jul 5;26(13):4104. doi: 10.3390/molecules26134104 (PMC8271896; doi:10.3390/molecules26134104)
Supplement: Supplementary file 1 [file molecules-26-04104-s001.zip › molecules-1266482-supplementary.pdf]

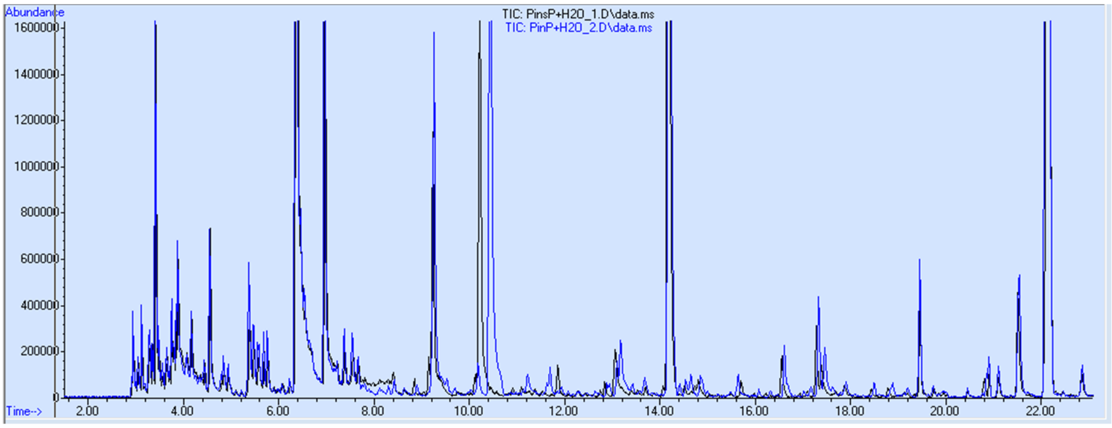


**Figure S1.** Comparison of chromatogram resolution of pea protein isolates with a 10 min equilibration Table 30. min equilibration time (blue).

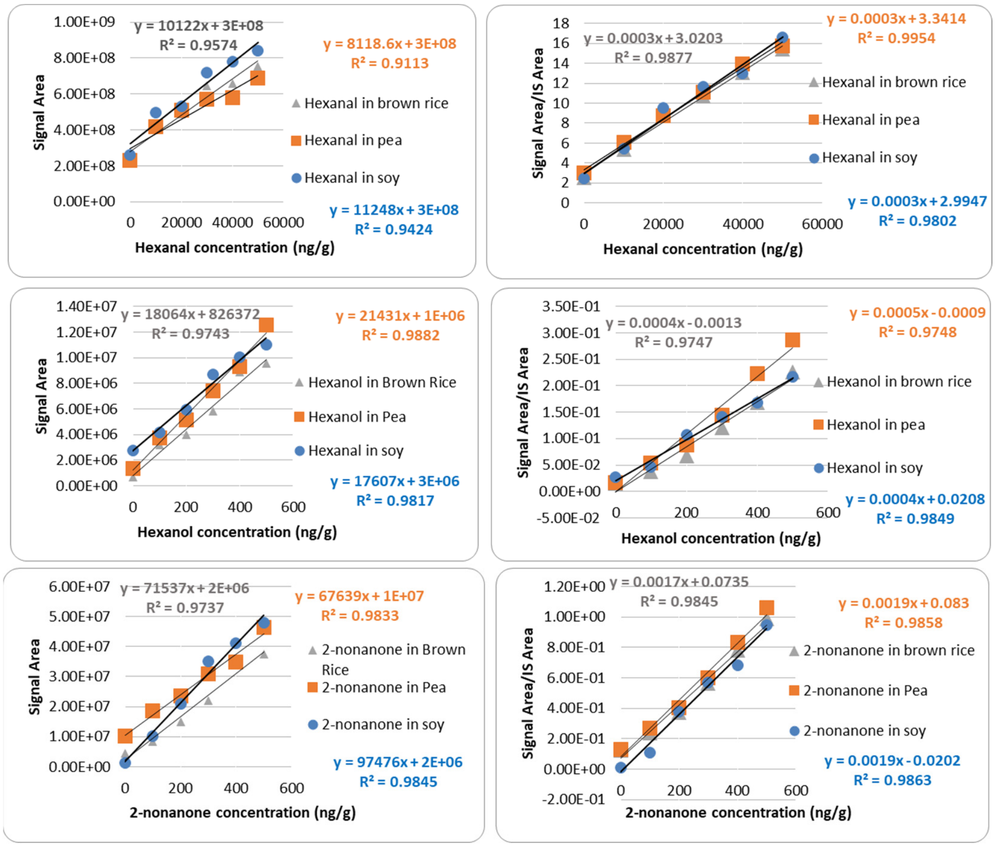


**Figure S2.** Calibration curves generated for hexanal, 1-hexanol and 2-nonanone in pea and soy protein isolates (calibration curves in red are for pea, and that in blue are for soy).

**Table S1.** Volatile profile of soy protein, allergen-free pea protein, and allergen-free brown rice protein.

| Protein | Compound Identification (Name, CAS, Formula, Molar Mass, OTV(Odour Threshold Value)) | Retention Time | Aroma | Concentration (in ng/g)[1] | Odor Activity Value OAV = ln (conc./OTV) |
|---|---|---|---|---|---|
| soy protein | Pentane, all isomers 78-78-4, 109-66-0, 463-82-1<br>$C_5H_{12}$<br>72.15<br>1290 | 2.94 | Gasoline-like | 60.36 ± 23.56 | Below Threshold |
| | n-Hexane<br>110-54-3<br>$C_6H_{14}$<br>86.17<br>1500 | 2.30 | Gasoline-like | 25.59 ± 13.80 | Below Threshold |
| | Octane, all isomers<br>111-65-9, 540-84-1, 86290-81-5<br>$C_8H_{18}$<br>114.22<br>660 | 3.40 | Green, minty, herbal, rosemary, cooling | 29.67 ± 15.60 | Below Threshold |
| | Furfural<br>98-01-1<br>$C_5H_4O_2$<br>96.08<br>2 | 4.12 | Chemical, beany, bready, malty | 108.76 ± 34.25 | 4.00 |
| | n-Valeraldehyde (Pentanal)<br>110-62-3<br>$C_5H_{10}O$<br>86.13<br>0.4 | 4.50 | Fermented, bready, fruity, nutty, berry | 89.12 ± 16.04 | 5.41 |
| | Hexanal<br>66-25-1<br>$C_6H_{12}O$<br>100.16<br>97 | 6.35 | Grassy, tallow, fatty, greeny | 1621.71 ± 159.69 | 2.82 |
| | 2-Heptanone (Methyl n-amyl Ketone)<br>110-43-0<br>$C_7H_{14}O$<br>114.18<br>140 | 9.18 | Soapy, sweet, mushroom | 72.63 ± 7.88 | Below Threshold |
| | Furan, 2-pentyl<br>3777-69-3<br>$C_9H_{14}O$<br>138.21<br>6 | 10.52 | Fruity, green, earthy, beany, vegetable, metallic | 2492.3 ± 199.4 | 6.03 |
| | 2-Octanone<br>111-13-7<br>$C_8H_{16}O$<br>128.21<br>50 | 13.01 | Soapy, fruity | 2.86 ± 2.51 | Below Threshold |
| | 3-Octen-2-one<br>4312-99-6<br>$C_8H_{14}O$<br>126.2 | 17.82 | Mushroom, mouldy, burnt | 12.03 ± 0.17 | 7.79 |
| | 1-Octen-3-ol<br>3391-86-4<br>$C_8H_{16}O$<br>128.2<br>1.08 | 19.43 | Mushroomy, earthy, burnt | 40.21 ± 9.61 | 3.62 |
| | Benzaldehyde<br>100-52-7<br>$C_7H_6O$<br>106.12 | 21.24 | Almond, burnt sugar, marchpane | 286.96 ± 25.56 | 5.25 |

| | | | | | |
|---|---|---|---|---|---|
| | 1.5 | | | | |
| pea protein | Octane, all isomers 111-65-9, 540-84-1, 86290-81-5 $C_8H_{18}$ 114.22 660 | 3.4 | Green, minty, herbal, rosemary, cooling | 25.48 ± 23.98 | Below Threshold |
| | n-Valeraldehyde (Pentanal) 110-62-3 $C_5H_{10}O$ 86.13 0.4 | 4.50 | Fermented, bready, fruity, nutty, berry | 90.65 ± 13.48 | 5.42 |
| | Decane 124-18-5 $C_{10}H_{22}$ 142.28 11 | 5.41 | Gasoline-like | 15.88 ± 15.91 | 0.37 |
| | Hexanal 66-25-1 $C_6H_{12}O$ 100.16 97 | 6.35 | Grassy, tallow, fatty, greeny | 1138.00 ± 297.3 | 2.46 |
| | Undecane 1120-21-4 $C_{11}H_{24}$ 156.31 620 | 7.61 | Green, minty, herbal, rosemary, cooling | 35.32 ± 62.71 | Below Threshold |
| | 2-Heptanone (Methyl n-amyl Ketone) 110-43-0 $C_7H_{14}O$ 114.18 140 | 9.18 | Soapy, sweet, mushroom | 25.64 ± 8.099 | Below Threshold |
| | Furan, 2-pentyl 3777-69-3 $C_9H_{14}O$ 138.21 6 | 10.52 | Fruity, green, earthy, beany, vegetable, metallic | 638.17 ± 49 | 4.67 |
| | Dodecane 112-40-3 $C_{12}H_{26}$ 170.33 110 | 11.42 | -- | 77.93 ± 59.84 | Below Threshold |
| | 2-Nonanone 821-55-6 $C_9H_{18}O$ 142.24 5 | 17.24 | Hot milk, soapy, greeny | 6.382 ± 0.6214 | 0.24 |
| | Nonanal 124-19-6 $C_9H_{18}O$ 142.24 1 | 17.35 | Fat, citrus, greeny | 31.82 ± 5.51 | 3.46 |
| | Benzaldehyde 100-52-7 $C_7H_6O$ 106.12 1.5 | 21.24 | Almond, burnt sugar, marchpane | 99.19 ± 4.22 | 4.19 |
| | 3, 5-Octadien-2-one 38284-27-4 $C_8H_{12}O$ 124.18 300 | 23.87 | Fruity, fatty, mushroom like | 48.71 ± 5.575 | Below Threshold |
| brown rice protein | Pentane, all isomers 78-78-4, 109-66-0, 463-82-1 $C_5H_{12}$ 72.15 1290 | 2.94 | Gasoline-like, sweet | 79.43 ± 4.97 | Below Threshold |

| | | | | |
|---|---|---|---|---|
| Heptane, all isomers 142-82-5, 590-35-2, 565-59-3 108-08-7, 591-76-4, 589-34-4<br>$C_7H_{16}$<br>100.2<br>410 | 3.12 | Petroleum-like | 167.98 ± 36.44 | Below Threshold |
| Acetone<br>67-64-1<br>$C_3H_6O$<br>58.08<br>20000 | 3.33 | Solvent, ethereal, apple, pear | 11.38 ± 1.89 | Below Threshold |
| Octane, all isomers<br>111-65-9, 540-84-1, 86290-81-5<br>$C_8H_{18}$<br>114.22<br>660 | 3.4 | Green, minty, herbal, rosemary, cooling | 652.63 ± 40.77 | Below Threshold |
| Butyraldehyde<br>123-72-8<br>$C_4H_8O$<br>72.11<br>0.3 | 3.6 | Pungent, cocoa, musty, green malty, bready | 137.19 ± 27.93 | 6.13 |
| n-Valeraldehyde (Pentanal)<br>110-62-3<br>$C_5H_{10}O$<br>86.13<br>0.4 | 4.5 | Fermented, bready, fruity, nutty, berry | 1837.33 ± 200.36 | 8.43 |
| Toluene<br>108-88-3<br>$C_7H_8$<br>92.13<br>21 | 5.3 | Sweet | 141.19 ± 17.08 | 1.91 |
| Decane<br>124-18-5<br>$C_{10}H_{22}$<br>142.28<br>11 | 5.41 | Gasoline-like | 89.78 ± 7.51 | 2.10 |
| Furfuryl Alcohol (2-furan methanol)<br>98-00-0<br>$C_5H_6O_2$<br>98.1<br>8000 | 5.69 | sweet, ether, alcohol | 53.81 ± 6.53 | Below Threshold |
| Hexanal<br>66-25-1<br>$C_6 H_{12}O$<br>100.16<br>97 | 6.35 | Grassy, tallow, fatty, greeny | 22590.24 ± 1643.70 | 5.45 |
| 2-n-Butyl furan<br>4466-24-4<br>$C_8H_{12}O$<br>124.18<br>51 | 7.12 | Mild, fruity, wine, sweet, spicy | 386.40 ± 30.64 | 2.03 |
| 5-undecene<br>764-96-5<br>$C_{11}H_{22}$<br>154.3<br>5 | 8.1 | pleasant, butter-like | 1410.36±296.77 | 5.64 |
| 2-Heptanone (Methyl n-amyl Ketone)<br>110-43-0<br>$C_7H_{14}O$<br>114.18 | 9.18 | Soapy, sweet, mushroom | 782.30 ± 81.94 | 1.72 |
| Isophorone<br>78-59-1<br>$C_9H_{14}O$ | 10.61 | sharp | 120.13 ± 104.12 | Below Threshold |

| | | | | |
|---|---|---|---|---|
| 138.2<br>190 | | | | |
| 2-Methylcyclohexanone<br>583-60-8<br>$C_7H_{12}O$<br>112.17<br>181 | 10.93 | | 147.85 ± 9.88 | Below Threshold |
| Pentanol, all isomers 71-41-0,75-85-4, 75-84-3, 123-51-3, 137-32-6, 584-02-1, 598-75-4, 6032-29-7, 30899-19-5, 94624-12-1<br>$C_5H_{12}O$<br>88.15<br>5.5 | 11.45 | Fruity | 1171.26 ± 31.66 | 5.36 |
| Diallylamine<br>124-02-7<br>$C_6H_{11}N$<br>97.16<br>2 | 12.75 | Disagreeable, refreshing | 113.44 ± 10.41 | 4.04 |
| 2-Octanone<br>111-13-7<br>$C_8H_{16}O$<br>128.21 | 13.01 | Soapy, fruity | 109.19 ±11.15 | 0.78 |
| Octanal<br>124-13-0<br>$C_8H_{16}O_{12}$<br>8.21<br>2 | 13.22 | Fatty, soap, lemon, green, orange, sweet | 981.91 ± 97.88 | 6.20 |
| 2-Heptenal (Butylacrolein)<br>18829-55-5<br>$C_7H_{12}O$<br>112.17<br>10 | 14.47 | Green type flavor | 134.37 ± 22.74 | 2.60 |
| 2-hexenal, 2-methyl<br>28467-88-1<br>$C_7H_{12}O$<br>112.17<br>17 | 14.87 | | 1716.45±112.42 | 4.62 |
| Dimethyl trisulfide<br>3658-80-8<br>$C_2H_6S_3$<br>0.001 | 15.46 | Sulfurous, onion, cooked, onion, savory, meaty | 273.51 ± 43.22 | 12.52 |
| 2-heptenal, 2-methyl<br>30567-26-1<br>$C_8H_{14}O$<br>13 | 15.64 | | 1717.48±204.72 | 4.88 |
| 1-Hexanol (n-Hexyl Alcohol)<br>111-27-3<br>$C_6H_{14}O$<br>102.18<br>2.4 | 15.66 | Resin, flowery, greeny | 102.04 ± 9.30 | 3.75 |
| Hexanethioic acid, S-methyl ester (S-Methyl hexanethioate)<br>2432-77-1<br>$C_7H_{14}OS$<br>146.25<br>0.3 | 17.06 | Fruity, ethereal, green, tropical, cabbage, cheesy, rancid, floral, green | 169.03 ± 21.49 | 6.33 |
| 2-Nonanone<br>821-55-6<br>$C_9H_{18}O$<br>142.24 | 17.24 | Hot milk, soapy, greeny | 94.02 ±12.38 | 2.93 |
| Nonanal<br>124-19-6 | 17.35 | Fat, citrus, greeny | 1129.05 ± 187.18 | 7.03 |

| | | | | |
|---|---|---|---|---|
| $C_9H_{18}O$<br>142.24<br>1 | | | | |
| 3-Octen-2-one<br>4312-99-6<br>$C_8H_{14}O$<br>126.2 | 17.82 | Mushroom, mouldy, burnt | 389.34 ± 46.50 | 11.26 |
| 1-TRIDECENE<br>2437-56-1<br>$C_{13}H_{26}$<br>182.35<br>1200 | 18.44 | Mild, pleasant. | 137.54 ± 14.33 | Below Threshold |
| 2-Octenal<br>2548-87-0<br>$C_8H_{14}O$<br>126.20<br>3 | 18.74 | Fatty | 450.75 ± 47.30 | 5.01 |
| 5-decanone (Methyl Octyl Ketone)<br>693-54-9<br>$C_{10}H_{20}O$<br>156.26 | 18.9 | Fermented | 34.98 ± 4.65 | Below Threshold |
| 1-Octen-3-ol,<br>3391-86-4<br>$C_8H_{16}O$<br>128.2<br>1.08 | 19.43 | Mushroomy, earthy, burnt | 1850.47±221.00 | 7.45 |
| 1-Heptanol,<br>111-70-6<br>$C_7H_{15}OH$<br>116<br>3 | 19.71 | Greeny | 421.46 ± 44.14 | 4.95 |
| 2,6-Dimethyl-5-heptenal<br>106-72-9<br>$C_9H_{16}O$<br>140.22<br>16 | 19.89 | -- | 125.05 ± 111.33 | 2.06 |
| Benzaldehyde<br>100-52-7<br>$C_7H_6O$<br>106.12<br>1.5 | 21.24 | Almond, burnt sugar, marchpane | 2798.73±367.53 | 7.53 |
| 2-heptenal, 2-propyl<br>532-27-4<br>$C_{10}H_{18}O$<br>154.59<br>16 | 21.61 | fruity | 121.15 ± 11.04 | 2.02 |
| Diisobutyl Ketone<br>108-83-8<br>$C_9H_{18}O$<br>142.24<br>103 | 21.89 | Peppermint | 263.69 ± 26.34 | 0.94 |
| n-Butyl Acrylate<br>141-32-2<br>$C_7H_{12}O_2$<br>128.17<br>0.3 | 22.58 | sweet, rancid, plastic | 118.36 ± 10.74 | 5.98 |
| Ethyl Amyl Ketone<br>541-85-5<br>$C_8H_{16}O$<br>128.21<br>5900 | 22.65 | solvent, sharp | 89.24 ± 56.36 | Below Threshold |
| 6-Undecanone (Diamyl ketone) 927-49-1<br>$C_{11}H_{22}O$<br>170.29<br>7 | 22.9 | | 94.74 ± 9.57 | 2.61 |

| | | | | |
|---|---|---|---|---|
| 1-Octanol<br>111-87-5<br>$C_8H_{18}O$<br>130.23<br>0.9 | 23.71 | Waxy green, orange, aldehydic, rose, mushroom | 397.56 ± 56.36 | 6.09 |
| 3, 5-Octadien-2-one<br>38284-27-4<br>$C_8H_{12}O$<br>124.18<br>300 | 23.87 | Fruity, fatty, mushroom like | 30.40 ± 2.82 | Below Threshold |
| 2-Octenal, 2-butyl<br>13019-16-4<br>$C_{12}H_{22}O$<br>182.30<br>3 | 24.25 | fruity, pineapple, green, aldehydic, sweet, ripe juicy flavor | 5906.10±807.06 | 3.49 |
| Valeric acid (Pentanoic acid)<br>109-52-4<br>$C_5H_{10}O_2$<br>102.13<br>940 | 28.8 | Acidic, sweaty, rancid | 225.04 ± 47.34 | Below Threshold |
| 2,4-Nonadienal<br>6750-03-4<br>$C_9H_{14}O$<br>138.21<br>0.5 | 28.9 | Fatty, melon, waxy, green, violet, leaf, cucumber, fruit, tropical, fruit, chicken, fat | 126.44 ± 22.71 | 5.53 |
| 1-Decene<br>872-05-9<br>$C_{10}H_20$<br>140.27<br>6450 | 33.79 | pleasant | 192.21 ± 29.43 | Below Threshold |
